# Immunonutrition in Early Life: Diet and Immune Development

**DOI:** 10.1155/2012/207509

**Published:** 2012-12-20

**Authors:** Francisco J. Pérez-Cano, Parveen Yaqoob, Rocío Martín, Margarida Castell Escuer, Cándido Juárez-Rubio

**Affiliations:** ^1^Department of Physiology, Faculty of Pharmacy, University of Barcelona, 08028 Barcelona, Spain; ^2^Department of Food and Nutritional Sciences, The University of Reading, Reading RG66AP, UK; ^3^Danone Research, Center for Specialised Nutrition, 6700 CA Wageningen, The Netherlands; ^4^Department of Immunology, Hospital Santa Creu i Sant Pau, 08041 Barcelona, Spain

## 1. Origins of Immunonutrition 

The belief that “you are what you eat” originated from Brillat-Savarin et al. (“tell me what you eat, and I will tell you who you are”) in the 1800s [1] was the starting point in the quest to understand the role of diet in almost every function in the body and in overall health. However, prior to the 1950s, there was little recognition of the relationship between nutrition and immune function. Scrimshaw and colleagues first described the bidirectional interaction between nutrition and infection [2]. This initial link between malnutrition and infective diseases has since evolved; not only do we now consider that diet is critical in maintaining optimal immune function, but it is widely accepted that almost all nutrients in the diet play a crucial role in maintaining an “optimal” immune response, and that both deficiency and excessive intakes can have negative consequences in terms of immune status and susceptibility to a variety of pathogens [3].

Dietary components with immunomodulatory potential include vitamins, minerals, polyphenols, dietary polyunsaturated fatty acids (PUFAs), and dietary components with the ability of modulating the gut microbiota (fibre, prebiotics, and probiotics). Although extensive research (experimental and clinical) demonstrates the properties of some of these dietary components, in many cases the exact mechanisms remain unclear. The impact of particular nutrients has been investigated by both epidemiological and intervention studies, but there are relatively few studies dealing with immune development in early life and in subjects with common immune-mediated diseases in early life (coeliac disease or asthma/allergies). 

## 2. Current Research in Immunonutrition

Half a century after the conceptualization of immunonutrition, the aim of this special issue is to shed some light on the mechanisms underlying the relationship between nutrition and immunity, especially in early life, when diet may play an important role in the immune development of the infant. This issue comprises two research articles and four review papers describing this complex relationship in humans and also in animal models, either in health or in disease. 

Human epidemiological data indicate that prenatal and early postnatal nutrition modulates the developing immune system. The confirmation of this epidemiologic association requires dietary intervention in human neonates, despite the ethical concerns and methodological considerations. Alternatively, or complementarily, animal studies are needed not only to elucidate the effect of nutrients or particular diets on immune function but also to understand mechanisms involved in these responses. In this context, the paper by F. J. Pérez-Cano et al. is a review of the different methodological approaches that can be used for early-life immunonutrition studies using animal models. It discusses the advantages and problems of using long versus short gestation period animal models and explores the use of suckling rats in these types of studies. The paper concludes that more complementary studies justifying specific animal models are required. 

The term immunonutrition has often been employed in the context of specific nutrients or combinations of nutrients used to treat surgical and critically ill patients who are at risk because of impaired or inappropriate immune activity. Historically, glutamine has been prominent in this area and in this issue, E. Briassouli and G. Briassoulis's review specifically addresses the use of glutamine in experimental and clinical studies in early life. They conclude that while there is promising data and plausible mechanisms, the evidence is still insufficient to allow recommendations to be made. 

Two papers in this issue deal with the influence of PUFAs on atopic and respiratory diseases in utero and during early life. Finally, two other papers examine novel strategies to modulate the exposure, development and remission of coeliac disease (CD). G. Mazzarella et al. evaluate the ability of transamidated gluten to maintain a gluten-free diet in CD patients in clinical remission, while T. Pozo-Rubio et al. review the influence of environmental factors on development of CD as well as strategies to induce or increase gluten tolerance. Interestingly, this paper suggests that modulation of the gut microbiota composition could be beneficial in this context.

## 3. Future Directions for Immunonutrition

Scientific expansion of global methodological approaches—the “omics” (genomics, transcriptomics, proteomics, metabolomics, microbiomics, etc.)—are beginning to influence research in immunonutrition and may prove to be useful tools in establishing mechanisms of action. However, nutrients are consumed as food; we cannot disregard the “matrix effect” and must recognize the importance of absorption, bioavailability, and so forth, in our approach. Moreover, the impact of genetic variability (nutrigenetics) and variation in the microbiome are still emerging and may in the future become increasingly important in the appropriate selection of subjects for experimental and clinical studies.

The design and the target period of a study, particularly when dealing with early development, can be crucial. In this sense, the fact that early life events (including gestational development) can have significant long-term effects is the basis of early programming of the immune system. Some of these events may involve dietary influences, either direct or indirect. Thus, the initial idea that “we are what we eat” has evolved in the last decade to “we are what our mother ate” [4]. Therefore, maternal nutrition and physiology are also relevant in this regard. However, many questions remain about how diet during development alters immune function in the long term. The use of appropriate experimental models and exploitation of “omics” techniques may help to unravel the answers.

*Francisco J. Pérez-Cano* *Parveen Yaqoob* *Rocío Martín* *MargaridaCastell Escuer* *Cándido Juárez-Rubio*
